# Chyluria With Coexisting Minimal Change Disease: An Uncommon Clinical Association

**DOI:** 10.7759/cureus.92800

**Published:** 2025-09-20

**Authors:** Regina J McPherson, Juan Ramon Santos Rivera, Nicholas V Manocchio, Mehak Sharma, Ilya Fonarov

**Affiliations:** 1 Medicine, Florida International University, Herbert Wertheim College of Medicine, Miami, USA; 2 Internal Medicine, Ponce Health Sciences University, Ponce, PRI; 3 Hospital-Based Medicine, Jackson Memorial Hospital, Miami, USA; 4 Internal Medicine, Florida International University, Herbert Wertheim College of Medicine, Miami, USA; 5 Internal Medicine, Jackson Memorial Hospital, Miami, USA

**Keywords:** chyluria, cloudy urine, lymphatic stasis, milky urine, minimal change disease (mcd)

## Abstract

Chyluria is a rare clinical entity defined by the presence of chyle in the urine, classically presenting as milky or cloudy urine. While typically associated with parasitic infections such as *Wuchereria bancrofti* in endemic regions, non-parasitic causes are less common and diagnostically challenging. We report the case of a 56-year-old male presenting with milky urine, dysuria, urinary hesitancy, and rectal discomfort. Urine triglyceride level was found to be >525 mg/dL (reference range <10 mg/dL). An extensive infectious and parasitic workup was negative. Renal biopsy revealed minimal change disease (MCD), a highly unusual finding in adult-onset chyluria. Although MCD does not cause chyluria, coexisting disease can contribute to urinary protein loss. This case highlights the importance of maintaining a broad differential diagnosis when evaluating urinary discoloration and emphasizes the rare intersection between glomerular disease and lymphatic-urinary communication. Early recognition of atypical presentations is essential to guide appropriate evaluation and prevent complications associated with chronic chyle loss.

## Introduction

Chyluria is an uncommon medical condition characterized by the presence of chyle in the urine, which gives it a distinctive milky or cloudy appearance. Chyle is an intestinal lymphatic fluid composed primarily of emulsified fats (triglycerides), proteins, and lymphocytes, which are normally transported via the thoracic duct into the venous system without entering the urinary tract [1,2]. The condition often arises due to abnormal communication between the lymphatic and urinary systems, typically at the level of the renal pelvis, ureter, or bladder, allowing chyle to leak into the urinary tract. This communication often results from rupture or obstruction of lymphatic vessels, leading to retrograde flow and fistulous formation between lymphatic and urinary structures [1,3].

Chyluria is broadly classified into parasitic and non-parasitic types. In endemic regions such as South Asia and parts of Sub-Saharan Africa, *Wuchereria bancrofti*, a mosquito-borne filarial parasite, is responsible for over 95% of confirmed parasitic cases [4]. In contrast, non-parasitic chyluria, more common in non-endemic areas, may result from penetrating injury or blunt trauma, infections such as tuberculosis and other fungal diseases can cause granulomatous inflammation and scarring of the lymphatic channels, tumors such as retroperitoneal lymphomas, congenital lymphatic malformations, or surgical disruption such as lymph node dissections involving the retroperitoneal space disrupting normal lymph flow [5]. Given this etiologic heterogeneity, diagnosing non-parasitic chyluria sometimes requires high-resolution imaging to identify the site and nature of lymphatic-urinary communications.

Diagnosis typically involves identifying urinary lipids, such as chylomicrons and triglycerides. Simple bedside methods, such as the ether clearance test or Sudan III staining, may support the diagnosis, and a urinary triglyceride concentration greater than 15 mg/dL is considered highly specific for chyluria [1].

While the most recognizable symptom is milky urine, patients may also present with dysuria, hematuria, flank pain, weight loss, malnutrition, and lower limb edema [2,3]. Systemic manifestations such as chills, weight loss, and peripheral edema may also be observed. In parasitic chyluria, additional features such as genital swelling, cellulitis, or abscesses may be present [1].

Chyluria is graded as mild, moderate, or severe based on symptom frequency, extent of calyceal involvement, and systemic manifestations. Mild cases typically involve intermittent milky urine without associated symptoms and are limited to a single calyx. Moderate chyluria includes intermittent or continuous milky urine with occasional associated symptoms and involvement of multiple calyces. Severe chyluria is defined by continuous milky urine with urinary retention, hematochyluria, systemic symptoms such as weight loss, and widespread involvement of the renal collecting system or ureter [1].

Chyluria management ranges from conservative to surgical, depending on the grade present. Some of the management approaches include dietary changes, analgesia, and anti-inflammatory agents. If the etiology is confirmed to be parasitic, pharmacologic therapy with agents such as diethylcarbamazine (DEC), ivermectin, or albendazole is recommended. DEC is the most commonly used drug and targets microfilariae, the larval stage. Albendazole, another commonly used drug, kills parasites by inhibiting microtubular proteins. Ivermectin, as a single dose, blocks the neurogenic transmissions of parasites [6]. Surgical interventions, including chylolymphatic disconnection, simple nephrectomy, or lymphovenous anastomosis, are reserved for refractory or advanced cases [6].

## Case presentation

**Figure 1:**
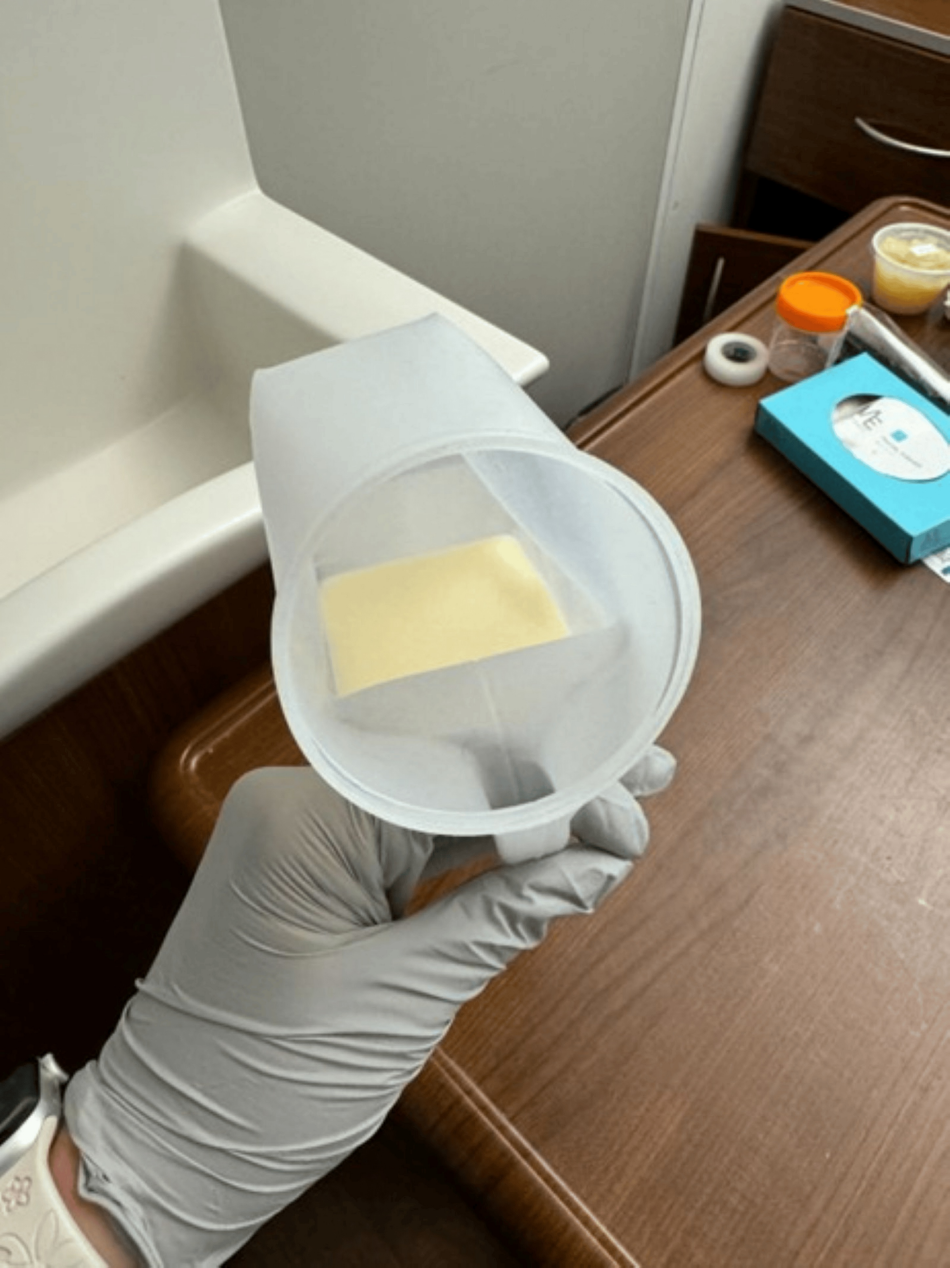
A top-down image of the same urine specimen, highlighting its creamy, homogeneous consistency. This visual is characteristic of chyluria and supports the diagnosis in the appropriate clinical context, differentiating it from other mimics such as pyuria, lipiduria, or phosphaturia.

**Figure 2:**
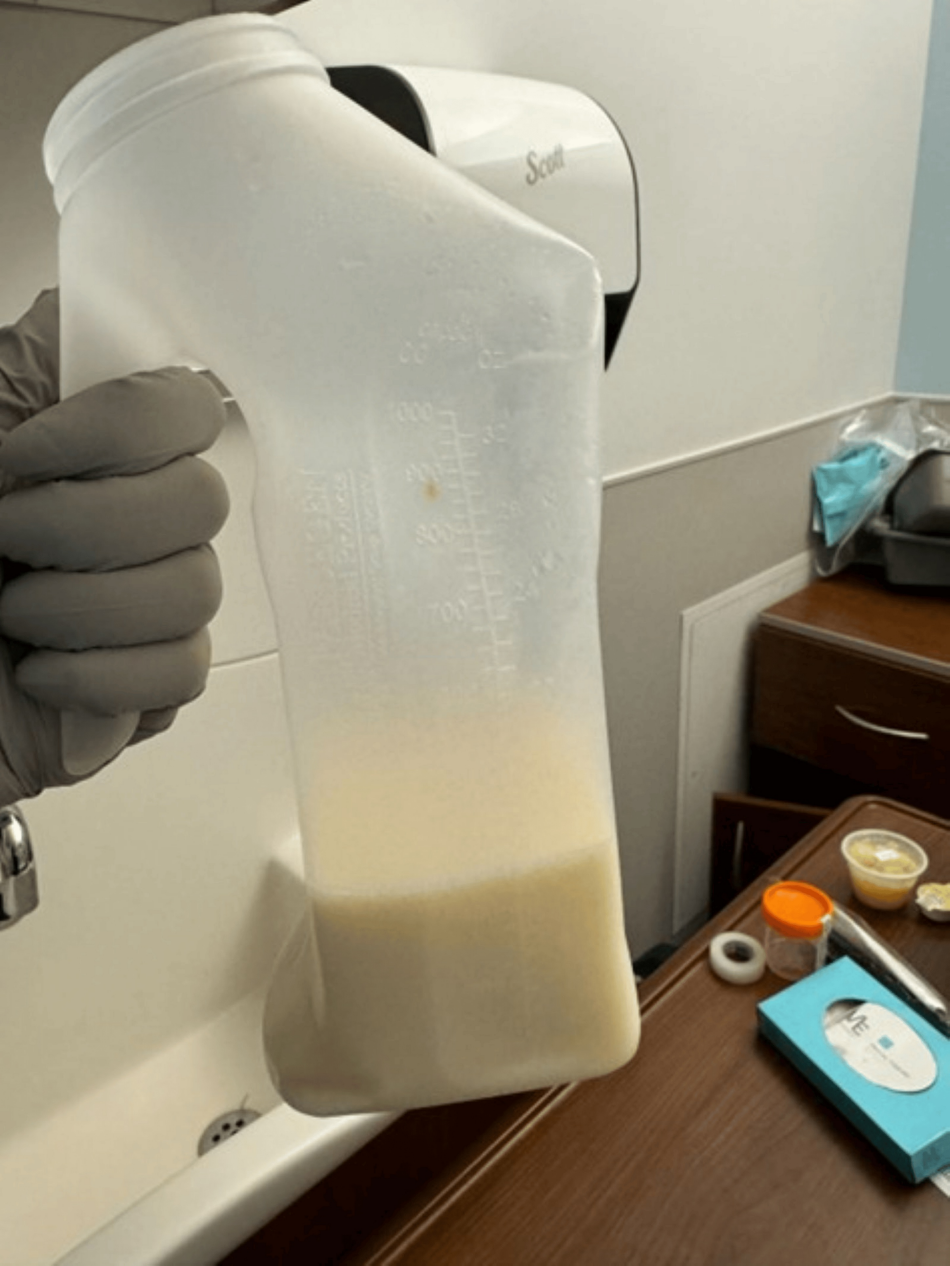
Bedside image of chylous urine collection container demonstrating the classic milky-white appearance of chyluria. The discoloration is due to the presence of lipid-rich lymphatic fluid (chyle) in the urine, resulting from an abnormal communication between the the lymphatic and urinary systems.

A 56-year-old male with no significant past medical or surgical history presented to the emergency room (ER) with complaints of white urine (Figures 1, 2) for about two weeks. He reported associated symptoms of dysuria, hesitancy, constipation, and rectal pain on defecation. He denied any nausea, vomiting, diarrhea, fevers, or chills. No abdominal pain or rectal bleeding was reported. In the emergency room, vital signs were within normal limits. Physical examination was unremarkable, including a digital rectal exam. Laboratory results were remarkable for elevated transaminases later found to be due to liver steatosis, elevated creatinine from baseline, hypoalbuminemia, and a urinalysis (UA) significant for proteins, glucose, and hematuria, and 24-hour urine protein resulted in >600 mg/24 hours. A urine triglyceride level was obtained and found to be greater than 525 mg/dL (Table 1). Other laboratory results were within normal limits. Human immunodeficiency virus (HIV) and hepatitis B and C screenings were negative.

**Table 1:** Significant laboratory values on admission.

Test	Result	Reference range
Creatinine	2.0 mg/dL	0.6–1.2 mg/dL
Blood urea nitrogen	41 mg/dL	7–20 mg/dL
Albumin	2.8 g/dL	3.5–5.0 g/dL
Aspartate transferase	65 U/L	10–40 U/L
Alanine transaminase	70 U/L	10–40 U/L
Glomerular filtration rate (estimated)	37 mL/minute/1.73m²	>60 mL/minute/1.73m²
24-hour urine protein	>600 mg/24 hours	<150 mg/24 hours
Urinalysis (UA) - protein	Positive	Negative
UA - glucose	Positive	Negative
UA - hematuria	Positive	Negative
Urine triglyceride level	>525 mg/dL	<10 mg/dL

Given the patient’s urinary findings and concern for glomerulonephritis, the nephrology team was consulted. A renal ultrasound was negative for hydronephrosis. He was seen by a colorectal surgeon who recommended a CT angiography (CTA) of the abdomen and pelvis with intravenous contrast and a colonoscopy for further evaluation of his rectal pain. No fistula or malignancy was noted on CTA. Colonoscopy was significant for a 4 mm tubular adenoma. An autoimmune workup with rheumatoid factor, anti-neutrophil cytoplasmic antibodies, and anti-double-stranded DNA was unrevealing. An infectious work-up was completed, including HIV, hepatitis panel, filaria, strongyloides, ova, and parasites, which returned negative. Urine electrophoresis, serum electrophoresis, free lambda kappa chains, and IgG level all returned negative. The elevated liver enzymes were due to liver steatosis. The patient remained stable while in the hospital.

The patient was considered to have stage 1 (mild) chyluria, and conservative management was recommended, including high fluid intake, a fat-restricted and high-protein diet. Given that no significant findings of fistulous process were identified on imaging, outpatient follow-up with the nephrology team was recommended. Following discharge, the nephrology team decided to proceed with a renal biopsy given the concern for an underlying glomerulonephritis. Renal biopsy was consistent with minimal change disease (MCD).

## Discussion

Chyluria is an uncommon condition characterized by the presence of chyle in the urine, leading to a milky or cloudy appearance. Etiologically, chyluria is broadly divided into parasitic and non-parasitic causes. In endemic regions, lymphatic filariasis, predominantly due to *Wuchereria bancrofti*, accounts for over 95% of parasitic chyluria cases [1,2]. In contrast, non-parasitic chyluria is more frequently reported in non-endemic settings and has been associated with trauma, malignancy, congenital lymphatic malformations, infections such as tuberculosis and fungal disease, and prior surgical interventions [3,4,7].

Presenting symptoms may include milky urine, dysuria, hematuria, weight loss, and flank pain. In this case, the patient presented with milky urine, dysuria, hesitancy, and was found to have microscopic hematuria. Although rectal discomfort was mentioned in the patient’s initial presentation, no anatomic or infectious cause was identified. It is possible the rectal discomfort was secondary to the patient’s constipation; this resolved by the time of hospital discharge.

The pathophysiology of chyluria involves lymphatic obstruction or disruption, which increases lymphatic pressure and leads to retrograde flow of chyle into the urinary tract through fistulous communications, typically at the level of the renal pelvis, ureter, or bladder [5]. In parasitic chyluria, chronic lymphatic inflammation and fibrosis promote lymphatic hypertension and fistula formation [6]. Non-parasitic cases are less well understood and often require advanced imaging such as CT urography or retrograde pyelography for diagnostic clarification [6]. Our patient was classified as having stage 1 chyluria, and conservative management was attempted.

Our patient denied any prior surgical procedures, penetrating trauma, or chronic infectious process. A comprehensive infectious and parasitic workup was negative. Ultimately, a renal biopsy performed due to significant proteinuria revealed MCD, an unexpected but critical finding in this context. MCD is a recognized cause of nephrotic syndrome, particularly in pediatric populations, but its presentation with chyluria in adults is exceedingly rare. Most adult cases of MCD present with generalized edema, hypoalbuminemia, and significant proteinuria without the presence of chyle in the urine [8,9]. In contrast, there are documented cases in which microfilaria have been identified on renal biopsy, directly linking parasitic infection to glomerular or urinary tract pathology [10]. Our patient demonstrated no such findings. We suspect our patient had concomitant coexisting MCD and chyluria, which are not pathophysiologically linked, as there is no established causal link between MCD and chyluria. In a case series by Sunder et al. of 18 patients with milky urine, 11 underwent biopsy, and only one patient was found to have MCD proven on biopsy, with the remainder having chyluria [11]. According to a case series by Kaul et al., large-scale studies are needed to better evaluate which patients with chyluria will benefit from a renal biopsy [12]. Further investigation is needed to determine which patients with white urine are at higher risk for glomerular disease and will benefit from a renal biopsy.

The initial management of chyluria typically entails conservative measures tailored to symptoms and severity, as was the case for our patient. Conservative treatment is likely to yield clinical improvement, as up to 50% of chyluria cases remit spontaneously. In mild cases, this includes high fluid intake, a fat-restricted and high-protein diet, and bed rest [1]. Triglyceride consumption should be minimized as triglycerides are absorbed directly into the portal system and bypass the lymphatics. Fat-soluble vitamins can be supplemented to prevent deficiency as well. Nutritional monitoring is warranted, as chronic chyluria can cause protein loss and anemia [13]. Sometimes, chylous clots can develop, causing colic. This can be managed with analgesics, anti-inflammatories, or bladder irrigation. The underlying causes should be addressed, with emphasis on non-parasitic etiologies in non-tropical climates.

Parasitic infection can be treated through pharmacological therapy with anti-helminthic options [1]. When suspecting a parasitic etiology, diethylcarbamazine (6 mg/kg/day for 21 days) is the first-line agent. In patients who cannot take diethylcarbamazine, ivermectin (6-12 mg ×1, repeat in three weeks) and albendazole (400-800 mg/day for 10-14 days) may also be used. Benzathine penicillin (1.2 million units weekly for 12 weeks) may be given for secondary prophylaxis of filariasis. All pharmacotherapy is given alongside conservative measures. There is some evidence that cholesterol-absorption inhibitors such as ezetimibe can be used to reduce lymphatic flow [1].

In our patient, ultrasound and CT scan imaging did not reveal a fistula that could be the source of chyluria. Our patient may need further testing and advanced imaging, such as MRI or cystoscopy, to locate the fistula causing chyluria if he fails conservative treatment.

For recurrent chyluria, despite conservative or medical therapy, when lymphatic fistula is established as the cause, endoscopic sclerotherapy should be considered as the first line to obliterate the lymphatic fistula. Agents such as 1% silver nitrate, 0.2% povidone-iodine can be used in this process. These agents induce chemical lymphangitis, which causes fibrosis to seal the fistula, giving immediate relief when the fistula is the root cause. Repeated sessions are often required, and treatment is typically given to one kidney at a time to avoid bilateral kidney injury. Sclerotherapy achieves a 70-80% clearance rate with fewer cases of long-term relapse compared to conservative care alone [1]. Minor side effects such as flank pain and transient hematuria are common but self-limiting.

Surgical intervention is reserved for those with refractory symptoms despite conservative and endoscopic therapy. In patients with recurrent ureteral obstruction by chylous clots, nutritional compromise, or failure of two or more courses of instillation, surgery is indicated. If surgery is planned, then lymphangiography can indicate the “site, size, and the number of fistulous communications” [1].

Chylolymphatic disconnection in combination with upper ureterolysis and nephropexy has been conducted with excellent success rates. Surgical treatment reports cure rates of 95-98% [1]. Postoperative rapid improvement of hypoalbuminemia and anemia reflects effective cessation of chyluria.

Clinically, the differential diagnosis for milky or cloudy urine is broad and includes several conditions that can mimic chyluria. Pyuria, typically associated with urinary tract infection, is characterized by leukocyturia and positive urine cultures [3,4]. There was no evidence of a urinary tract infection in our patient. Phosphaturia is a benign and transient cause of cloudy urine that clears upon acidification, for example, with vinegar [3]. Lipiduria, often encountered in nephrotic syndrome, is identified by the presence of oval fat bodies and a classic “Maltese cross” appearance on polarized microscopy [8]. Although rare, fungal infections can lead to granulomatous lymphatic obstruction and present similarly to chyluria [7]. In older patients or those with systemic signs, malignancies, particularly retroperitoneal tumors or lymphomas, must also be considered, as they may compress or invade lymphatic structures [4].

The workup for chyluria should include urinalysis, urine triglyceride concentration, ether clearance testing, and, when appropriate, renal imaging or biopsy. A urinary triglyceride level >15 mg/dL and the presence of chylomicrons are highly specific for chyluria [3]. Of note, our patient presented with a urine triglyceride level of > 525 mg/dL (reference range <10 mg/dL). Prompt recognition and diagnosis are essential, as prolonged chyle loss can result in protein-energy malnutrition, electrolyte disturbances, lymphopenia, and fat-soluble vitamin deficiencies [9].

From a public health perspective, parasitic chyluria remains an underrecognized burden in endemic regions, often linked to inadequate sanitation and poor vector control. Public health strategies, including mosquito abatement, education, and access to anti-filarial therapy, remain vital to disease prevention [1,6]. However, this case highlights the importance of maintaining a broad differential and considering non-parasitic causes in patients presenting from non-endemic regions or with negative infectious evaluations.

## Conclusions

Chyluria remains a clinically and socially significant condition due to its diverse etiologies. Although often benign, it requires careful evaluation to distinguish between parasitic and non-parasitic causes, particularly in non-endemic settings. In this case, nephrotic syndrome and chyluria were coexisting. Nephrotic syndrome can be explained by MCD found on renal biopsy, while chyluria remains unexplained and will need further investigation. The coexistence of MCD and chyluria in this case is not pathophysiologically linked but considered incidental. This case highlights the importance of maintaining a broad differential diagnosis and demonstrates that persistent proteinuria in a patient with chyluria should prompt consideration of glomerular disease, particularly when infectious and structural evaluations do not reveal a direct cause. Further investigation is needed to determine which patients with white urine are at higher risk for glomerular disease and will benefit from a renal biopsy.
